# DNA damage and oxidative stress response to selenium yeast in the non-smoking individuals: a short-term supplementation trial with respect to *GPX1* and *SEPP1* polymorphism

**DOI:** 10.1007/s00394-015-1118-4

**Published:** 2015-12-10

**Authors:** E. Jablonska, S. Raimondi, J. Gromadzinska, E. Reszka, E. Wieczorek, M. B. Krol, A. Smok-Pieniazek, M. Nocun, M. Stepnik, K. Socha, M. H. Borawska, W. Wasowicz

**Affiliations:** 1 Department of Toxicology and Carcinogenesis, Nofer Institute of Occupational Medicine, St. Teresy 8 Street, 91-348 Lodz, Poland; 2 Division of Epidemiology and Biostatistics, European Institute of Oncology, Via Ripamonti 435, Milan, Italy; 3 Department of Bromatology, Medical University of Bialystok, Mickiewicza 2D Street, 15-222 Białystok, Poland

**Keywords:** Selenium, Selenoproteins, Oxidative stress, DNA damage, Gene expression, Selenium supplementation

## Abstract

**Purpose:**

Selenium, both essential and toxic element, is considered to protect against cancer, though human supplementation trials have generated many inconsistent data. Genetic background may partially explain a great variability of the studies related to selenium and human health. The aim of this study was to assess whether functional polymorphisms within two selenoprotein-encoding genes modify the response to selenium at the level of oxidative stress, DNA damage, and mRNA expression, especially in the individuals with a relatively low selenium status.

**Methods:**

The trial involved 95 non-smoking individuals, stratified according to *GPX1* rs1050450 and *SEPP1* rs3877899 genotypes, and supplemented with selenium yeast (200 µg) for 6 weeks. Blood was collected at four time points, including 4 weeks of washout.

**Results:**

After genotype stratification, the effect of *GPX1* rs1050450 on lower GPx1 activity responsiveness was confirmed; however, in terms of DNA damage, we failed to indicate that individuals homozygous for variant allele may especially benefit from the increased selenium intake. Surprisingly, considering gene and time interaction, *GPX1* polymorphism was observed to modify the level of DNA strand breaks during washout, showing a significant increase in *GPX1* wild-type homozygotes. Regardless of the genotype, selenium supplementation was associated with a selectively suppressed selenoprotein mRNA expression and inconsistent changes in oxidative stress response, indicating for overlapped, antioxidant, and prooxidant effects. Intriguingly, DNA damage was not influenced by supplementation, but it was significantly increased during washout.

**Conclusions:**

These results point to an unclear relationship between selenium, genotype, and DNA damage.

**Electronic supplementary material:**

The online version of this article (doi:10.1007/s00394-015-1118-4) contains supplementary material, which is available to authorized users.

## Background

Selenium (Se) is an essential trace element, the importance of which for human health is indicated by the presence of at least 25 selenoproteins containing the element in a form of selenocysteine (Sec), the twenty-first proteinogenic amino acid. The unusual uniqueness of selenium stems from the fact that Sec is incorporated into selenoprotein polypeptide chain cotranslationally, driven by UGA mRNA codon in the presence of a specific molecular machinery, including Sec-tRNA, Sec insertion sequence (SECIS) element, and protein factors such as SECIS-binding protein (SBP2) and Sec-specific translation elongation factor (eEFSec), which all together allow for insertion of Sec instead of premature termination of biosynthesis (as UGA usually serves as the termination codon) [1, 2]. The uniqueness of selenium is also reflected by its biological activity, in terms of which the element is most often described as “double-edge sword,” “essential poison,” or “two-faced element” [3]. That is due to high redox properties—Se exerts both nutritional and toxic activities within a relatively narrow range of doses, highlighted by a relatively small difference between the recommended daily intake (55 µg or 70 µg in USA and Europe, respectively) and the estimated upper tolerable limit (300, 400, or 450 µg/day according to different sources) [4–6]. Most of the scientific interest related to Se is associated with its anticancer properties, which have been suggested based on numerous in vitro and in vivo studies as well as on some, although not all, human observational studies and human long-term supplementation trials [7, 8]. Mechanism of anticancer activity of Se remains elusive, but it is generally considered that it results both from antioxidant- and redox-related properties of Se, exerted at a nutritional level and linked mainly to the activity of selenium-dependent enzymes (selenoproteins with catalytic activity), as well as from its prooxidant effects, observed at supranutritional intake and associated with the activity of low molecular weight Se compounds [9–12]. However, in the recent years, the hypothesis on cancer protective activity of Se has been seriously undermined by the results of a large, randomized double-blind prospective study, i.e., SELECT, which has been conducted to test whether Se (as selenomethionine) and vitamin E (as alpha-tocopherol) may reduce the risk of prostate cancer in men, and which has finally failed to indicate any protective effects of the element, either alone or in the combination with vitamin E [13]. Additionally, some concern has been paid to Se supplementation in terms of its possible diabetogenic effects, adding more uncertainty to assessing the relationship between selenium and human health [14, 15].

On the basis of several gene association studies, pointing to the importance of genetic polymorphism in the risk of human cancer as well as other diseases, it has been hypothesized that biological activity of Se in the human body may be modified by genetic polymorphism of selected selenoproteins [16, 17]. Human selenoproteins cover many biologically important functions like those related to antioxidant defense system, thyroid hormone metabolism, and redox balance maintenance, all of which are driven mainly by redox-related selenoproteins, including five glutathione peroxidases (GPx1, GPx2, GPx3, GPx4, GPx6), three iodothyronine deiodinases (DI1, DI2, DI3), three thioredoxin reductases (TRxR1, TRxR2, TRxR3), or methionine-R-sulfoxide reductase (MsrB1, known also as SelR or SelX). Selenoprotein P (Sepp1) is the only selenoprotein which possesses multiple Sec residues and which is mainly responsible for Se transport within the body, but has been also shown to exert catalytic and antioxidant activities [18]. Functions of other selenoproteins are specifically linked to the selenoprotein synthesis (selenophoshate synthetase 2—SPS2), muscle development (selenoprotein N—SelN) or endoplasmic reticulum function (15-kDa selenoprotein—Sep15, selenoprotein S—SelS, selenoprotein K—SelK), whereas functions of many other (like selenoprotein H—SelH, selenoprotein I—SelI, selenoprotein M—SelM, selenoprotein O—SelO, selenoprotein W—SelW, selenoprotein T—SelT, or selenoprotein V—SelV) still remain unknown [19, 20]. It has been demonstrated that some polymorphic variants in genes encoding for selected selenoproteins, including *GPX1* (glutathione peroxidase 1), *GPX4* (glutathione peroxidase 4), *SEPP1* (selenoprotein P), *SELS* (selenoprotein S), and *SEP15* (15-kDa selenoprotein*),* have functional significance and may influence expression or activity of the protein either at the transcription, translation, or posttranslational level, thus explaining their association with the altered risk of cancer at several sites [17].

Despite the commonly appreciated approach to include genetic profile in the design of intervention trials or in the interpretation of study results, only few authors have followed this direction, and those who have, focused only on single, specific end points linked mainly to oxidative stress markers and/or DNA damage [21–25]. Notably, the genotype modifying effects are more likely to be observed within the subjects with a relatively low Se status [24]. Thus, the aim of this study was to analyze the potential influence of two functional single-nucleotide polymorphisms (SNPs), present within the coding region of two selenoprotein genes: *GPX1* rs1050450 (Pro198Leu) and *SEPP1* rs3877899 (Ala234Thr), on the multilevel biological response to Se supplementation. Due to a low dietary intake of Se in the Polish population [26], individuals from Poland who constituted the study group seemed to be relevant to investigate the possible gene–selenium interactions. Biomarkers of relevance included markers of Se status (plasma Se and Sepp1 concentrations), markers of oxidative stress (activity of antioxidant enzymes in plasma or erythrocytes, plasma lipid peroxidation, oxidative burst in the whole-blood leukocytes), DNA strand breaks, DNA oxidation, and mRNA expression of selected encoding selenoproteins or related genes (*GPX1*, *GPX4*, *TRXR1*, *SEP15*, *SEPP1*, *SELS*, *SELW*, *SBP2*).

## Methods

### Study design

By means of advertisement in different public places, 517 residents of Lodz in the age 18–60 were recruited for the study. Questionnaire data and 4 mL of fasting blood were collected from the subjects, followed by DNA isolation and prospective genotyping for *GPX1* rs1050450 and *SEPP1* rs3877899. In a whole group of 517 recruited subjects, *GPX1* allelic variants were in Hardy–Weinberg equilibrium (*p* = 0.582), and the genotype distribution was as follows: 46, 43, and 11 % for ProPro, ProLeu, and LeuLeu, respectively. *SEPP1* genotype frequencies were 60, 32, and 7.5 % for AlaAla, AlaThr, and ThrThr, respectively, with a slight deviation from Hardy–Weinberg equilibrium (*p* = 0.013). The exclusion criteria for the supplementation trial included current smoking, chronic diseases such as cancer, diabetes, or cardiovascular disease, BMI higher than 35 as well as incomplete data for genetic analyses. Altogether 137 subjects were excluded. Further selection of the remaining 380 volunteers was based on the approximate major and minor allele distribution in order to obtain an as high as possible number of individuals with rare genotypes (*GPX1* Leu/Leu and *SEPP1* Thr/Thr) and to increase statistical power of comparison tests. Namely, all the subjects with rare genotypes were asked to participate in the supplementation trial, whereas subjects with common alleles were matched for age and BMI. Altogether 95 subjects agreed to participate in the trial. The study group characteristics are presented in Table 1. Genotype distribution is shown in Table 2, whereas Figure S1 presents the numbers of subjects with different genotype combinations. The selected 95 subjects, including 43 men and 52 women at the mean age of 35.6 years, were receiving 200 µg of selenium in a form of selenium yeast for 6 weeks. Se yeast tablets were obtained commercially, and all were from the same batch. During the supplementation trial, as well as during the washout period, the participants were asked not to take any supplements containing vitamins and selenium or other elements. Fasting blood was collected during the study at four time points: before the supplementation assigned as baseline (the first day of the trial, before taking the first tablet), after 2 and 6 weeks of supplementation, and after 4 weeks of the washout period. Blood samples were collected from each participant in two heparin tubes and two EDTA tubes. The material collected in the first heparinized tube (7.5 mL) was fractioned by centrifugation into plasma, buffy coat, and erythrocytes and frozen at −20 °C until biochemical analyses. Whole blood (4.5 mL) from the second heparin tube was used for oxidative burst assessment, which was performed at the day of blood collection (this analysis was performed only at two time points: at baseline and after 2 weeks of supplementation). EDTA whole blood (1.5 mL) was used for leukocyte lysate preparation, which was subsequently stored at −70 °C until mRNA isolation (for gene expression analysis). Whole blood (2.7 mL) from the second EDTA tube was used to prepare agarose slides for the comet assay (this analysis was performed at three time points: at baseline, after 6 weeks of supplementation, and after 4 weeks of the washout period). Along with the blood collection, detailed questionnaire data were collected each time, concerning current health status (well-being with respect to adverse health effects), intake of medications, dietary supplements, herbs or pharmacological treatment with hormones, antibiotics, statins, and other. All the analyses, apart from the oxidative burst assessment, were conducted after completing the study, and all the samples were blinded in terms of genotype and the time point of their collection. All the study participants gave written informed consent, and the subjects who were enrolled for the supplementation trial received detailed, written, and oral information about the trial. The study was approved by the Local Ethics Committee (Ethical Institutional Review Board at the Nofer Institute of Occupational Medicine, Lodz, Poland).

**Table 1:** Baseline characteristics of the study group

Characteristics	*N* (%)	Mean ± SD (range)
All	95 (100)	
Males	43 (45)	
Females	52 (55)	
Age (years)	95 (100)	35.6 ± 10.8 (18-60)
<30	31 (32.6)	
31–40	41 (43.2)	
41–50	9 (9.5)	
51–60	14 (14.7)	
BMI (kg/m^2^)	95 (100)	23.8 ± 3.1 (17.9-34.9)
<25	68 (71.6)	
25–30	23 (24.2)	
>30	4 (4.2)	
Smoking		
Current	0 (0)	
Ever	25 (26.3)	
In the past 5 years	10 (9.5)	
Never	70 (73.7)	
Passive smoking		
Yes	25 (26.3)	
No	70 (73.7)	
Alcohol consumption		
Never	5 (5.3)	
Less than 1 day per month	30 (31.6)	
Less than 1 day per week	34 (35.8)	
Up to 2 days per week	25 (26.3)	
3–5 days per week or more	1 (1.1)	
Vitamin or mineral supplements use		
Yes, sporadically	34 (35.8)	
Yes, regularly	23 (24.2)	
No	38 (40.0)	
Selenium containing supplements use in the past 6 months		
Yes	5 (5.3)	
No	90 (94.7)

**Table 2:** Genotype distribution for *GPX1* rs1050450 and *SEPP1* rs3877899 polymorphisms and baseline characteristics according to genotype

Genotype	*N* (%)	Age, years (median and range)	Sex (females/males)	BMI (median and range)	Plasma Se, µg/L (median and range)	Plasma Sepp1 ng/mL (median and range) *
*GPX1* rs1050450						
Pro/Pro	39 (41 %)	33.0 (18.0–60.0)	1.29 (22/17)	22.9 (18.0–33.1)	63.3 (34.3–109.1)	3.9 (0.9–26.4)
Pro/Leu	35 (37 %)	37.0 (18.0–58.0)	1.06 (18/17)	23.7 (17.9–31.1)	62.7 (35.8–103.2)	4.2 (1.3–36.0)
Leu/Leu	21 (22 %)	34.0 (18.0–58.0)	1.33 (12/9)	24.6 (20.1–34.9)	62.5 (37.2–96.2)	3.6 (1.0–6.4)
*SEPP1* rs3877899						
Ala/Ala	46 (48 %)	33.0 (20.0–58.0)	0.92 (22/24)	23.4 (18.7–31.1)	66.3 (37.2–106.1)^a^	3.1 (1.0–26.9)
Ala/Thr	29 (31 %)	33.0 (18.0–58.0)	1.90 (19/10)	22.9 (17.9–27.7)	68.9 (34.3–96.2)^a^	4.2 (0.9–36.0)
Thr/Thr	20 (21 %)	36.5 (18.0–60.0)	1.22 (11/9)	23.7 (18.9–34.9)	52.0 (35.8–109.1)^a^	4.3 (1.1–23.4)

* Skewed data; for these parameters, *p* values (one-way ANOVA) were calculated for log-transformed data

^a^ *p* for linear trend = 0.04

### DNA isolation and SNP genotyping

DNA was isolated from buffy coat, using QIAamp DNA Blood Mini Kit (Qiagen, Hilden, Germany). Allelic discrimination for the two studied polymorphisms was performed using the real-time PCR method and CFX96™ Real-Time PCR Detection System (Bio-Rad, Hercules, CA, USA). *GPX1* rs1050450 genotyping was conducted using the high-resolution melt curve (HRM) technique. Oligonucleotide sequences for PCR primers, designed by Beacon Designer™ (PREMIER Biosoft, Palo Alto, CA, USA), were as follows: 5′-GCCGCTTCCAGACCATTG-3′ (forward) and 5′-GGTGTTCCTCCCTCGTAG-3′ (reverse). Data analysis was performed using Bio-Rad CFX Manager and Bio-Rad Precision Melt Analysis Software. Identification of particular genotypes recognized by HRM was based on the comparison with the method introducing specific fluorescent probes, as described previously [27]. For *SEPP1* rs3877899 analysis, the TaqMan^®^ SNP Genotyping Assay (C_8709053_10, Life Technologies, Life Technologies, Carlsbad, CA, USA) was used. The call rate for two SNPs was >99 %, and the concordance of the blinded QC (*n* = 12) was 100 %.

### mRNA isolation, cDNA synthesis, and gene expression

WBC lysates and mRNA isolation were performed using the QIAamp RNA Blood Mini Kit (Qiagen, Hilden, Germany), and reverse transcription was performed using the QuantiTect Kit (Qiagen, Hilden, Germany). Both cDNA synthesis and real-time PCR were conducted in the Light Cycler 96 Real-Time PCR System (Roche, Indianapolis, IN, USA). cDNA was stored at −20 °C until gene expression experiments. Primers’ sequences and amplicons’ sizes are presented in Table S1. Real-time PCRs were carried out in three replications for each sample. 20 µL reaction mixture contained 1 μL of cDNA, 10 μL of 2x FastStart SYBR Green Master (Roche, Indianapolis, IN, USA), 20 pmol of each primer, and nuclease-free water (Qiagen, Hilden, Germany). PCR conditions were as follows: polymerase activation at 95 °C for 10 s, followed by 45 cycles: denaturation at 95 °C for 15 s, annealing at 60 °C for 45 s, and polymerization at 72 °C for 45 s. Inter- and intra-assay variations, as determined by three occasional, intra-experimental runs of five cDNA triplicates, were below 12 and 10 %, respectively. PCR efficiencies were calculated using five dilutions of pooled cDNA, consisting of randomly selected samples. As confirmed by the initial data analysis, expression of the reference gene (*GAPDH*) was stable under experimental conditions. Normalized relative quantification of the target genes’ expression was evaluated including reaction efficiency correction, by the use of the qbasePLUS software, version: 2.3) (Biogazelle NV, Zwijnaarde, Belgium).

### Plasma selenium concentration

Plasma sample used for determination of selenium concentration was initially diluted with Triton X-100 (0.2 %, Sigma-Aldrich, St. Louis, MO, USA) in a proportion 1:1. Concentration was assessed using Zeeman flameless atomic absorption spectrometry (instrument Z-5000, Hitachi) with graphite tube and palladium/magnesium matrix modifier (Pd—1500 ppm, Mg—900 ppm). The limit of detection was 1.4 µg/L. The certified reference material Seronorm Trace Elements Serum (SERO AS, Billingstad, Norway) was used for the quality control assessment. The accuracy of the method and coefficient of variance (CV) were 1.3 and 4.7 %, respectively.

### Plasma selenoprotein P concentration

Sepp1 concentration was determined in plasma using the immunochemical Sandwich ELISA test (USCN Life Science Inc kit, Hu, China). In brief, plasma samples were diluted 500–1000 times and added to the plate wells with biotin-conjugated polyclonal antibody specific for Sepp1. Next, avidin-conjugated horseradish peroxidase was added, and after incubation with TMB (3,3′,5,5′-tetramethylbenzidine), the substrate solution color change was observed proportionally to Sepp1 concentration. The reaction was terminated with sulfuric acid solution, and color intensity was measured at a wavelength of 450 nm. For each test, a standard curve was determined (range 0.78–50 ng/mL), and Sepp1 concentration was calculated after plotting logarithmic curve. Intra-assay and inter-assay variations were 8.0 and 12.7 %, respectively.

### Activity of antioxidant enzymes

Spectrophotometric methods were used to analyze blood compartments for the activity of GPx1, GPx3, SOD1, and Cp. The activity of glutathione peroxidases was determined in erythrocytes (GPx1) and plasma (GPx3) using the method of Paglia and Valentine [28] with t-butyl hydroperoxide as a substrate and following the rate of NADPH oxidation by the coupled reaction with glutathione reductase. The rate of decrease in the absorbance, being proportional to the GPx activity, was read at a wavelength of 340 nm. The activity of SOD1 was determined in erythrocytes by the use of the method of Beauchamp and Fridovich [29], which relies on the inhibition by SOD1 of the reduction of nitro blue tetrazolium (NBT) by xanthine and xanthine oxidase. The concentration of the reduced form of NBT was measured at a wavelength of 540 nm. The oxidase activity of Cp was determined in plasma according to the method described by Sunderman and Nomoto [30], with a PPD (*p*-phenylenediamine) as a substrate. The absorbance of the oxidation product was read at a wavelength of 535 nm. The activity of Cp was expressed as the amount of product formed per minute per 1 L of plasma. All the absorbance values were read using the Unicam UV4 UV/Vis spectrophotometer (Cambridge, UK).

### Total antioxidant capacity

The total antioxidant capacity of plasma was determined calorimetrically by the use of the Antioxidant Assay Kit (Cayman Chemical Company, Ann Arbor, MI, USA). Absorbance measurement was taken on a spectrophotometer MultiScan GO (Thermo Scientific, Waltham, MA, USA).

### Lipid peroxidation

Plasma concentration of thiobarbituric acid-reactive substances (TBARS) was determined using the spectrofluorometric method, optimized by Wasowicz et al. [31]. TBA-reactive compounds were extracted to butanol. The value of fluorescence of butanol layer was read at an excitation wavelength of 525 nm and an emission wavelength of 547 nm, using the PerkinElmer Luminescence Spectrometer LS50B (Norwalk, Ct, USA).

### Oxidative burst

Oxidative burst (generation of reactive oxygen species, ROS) was measured using flow cytometry and fluorescent labeling with 2′,7′-dichlorodihydrofluorescein diacetate (DCFH_2_-DA, Sigma-Aldrich, St. Louis, MO, USA). The fluorescence was read in the FACSCanto II flow cytometer (BD Biosciences, San Jose, CA, USA) and expressed as the mean fluorescent intensity (MFI). The final results were expressed as the MFI ratio, calculated as the ratio of PMA stimulated cells MFI to MFI of basal ROS production. In each sample, at least 20,000 cells were examined.

### DNA damage

DNA damage, including the strand breaks (SB) and alkali-labile sites (ALS), was assayed in whole blood using alkaline single-cell gel electrophoresis (SCGE; comet assay) method as described by Singh et al. [32] and modified by Mc Kelvey–Martin et al. [33]. One aliquot of whole blood was mixed with nine aliquots of RPMI-1640 medium and 10 aliquots of 2 % (in PBS) molten agarose type VII (low gelling temperature). The mixture was then spread on a slide earlier covered with agarose type I (low EEO) at 1 % concentration in water and dried. The cells embedded in the agarose gel were lysed in cold lysing solution (2.5 M NaCl, 100 mM Na2EDTA, 10 mM Tris base, pH 10, with 1 % Triton X-100 added just before use) at 4 °C for 1 h. Subsequently, DNA was unwound in an alkaline electrophoresis buffer (1 mM Na2EDTA, 300 mM NaOH, pH 13) for 20 min and electrophoresed in the same alkaline conditions (30 min, 25 V, 300 mA, 1.04 V/cm). The gels were then neutralized by rinsing three times with 0.4 M Tris buffer (pH 7.5), and the slides were dried for storage. In parallel analyses, oxidatively generated damage to DNA bases was additionally identified as formamidopyrimidine glycosylase (FPG)-sensitive sites using modified comet assay as described earlier by Collins et al. [34]. FPG enzyme was purchased from New England Biolabs (Hithchin, UK). After lysis, the slides were washed three times with an enzyme buffer (0.1 M KCl, 0.5 mM Na2EDTA, 40 mM HEPES–KOH, 0.2 mg/ml bovine serum albumin, pH 8) and incubated with FPG at 2.7 U/mL in this buffer (kept at −80 °C) for 30 min at 37 °C. The slides were then electrophoresed and neutralized as described above. Finally, the slides were stained with 5 μg/mL DAPI, and 50 cells from each slide were analyzed using an Olympus fluorescence microscope (a BX40 instrument; Olympus, Tokyo, Japan) equipped with an image analysis system (Comet IV, Perceptive Instruments, UK). For each participant, four slides were prepared simultaneously: two for assessment of DNA strand breakage and the other two, which included also the FPG treatment, for the assessment of total DNA damage (i.e., DNA strand breakage and oxidatively generated DNA damage). Respective DNA damage was inferred based on the relative amount of DNA in the comet tail (henceforth referred to as % tail DNA) obtained via computer-aided image analysis. DNA oxidation damage was expressed as the difference between the total DNA damage and DNA strand breaks, i.e., “net FPG-sensitive sites.”

### Statistical analysis

Data normality was assessed using Shapiro–Wilk test. In the case of abnormal distributions, an analysis was performed on the log-transformed values. Baseline differences between males and females were assessed by the use of the *t* test, while correlations among baseline parameters and individuals’ characteristics were evaluated by the Pearson correlation coefficient. Differences in the distribution of baseline parameters (age, BMI, baseline selenium, and plasma Sepp1 concentration) according to *GPX1* rs1050450 and *SEPP1* rs3877899 genotypes were evaluated by one-way ANOVA. Repeated measures analysis of covariance (ANCOVA, MANCOVA) was carried out to test the effects of particular genotype or genotypes interaction on the studied biological parameters. With MANCOVA analysis, parameters measured at different time points were considered as dependent variables, while genotypes and covariates were included in the models as independent variables. Covariates included age, sex, BMI, and baseline selenium. The effect of Se supplementation (time effect/within subjects effect) as well as the effect of interaction between genotype and Se supplementation was assessed using MANCOVA test methods (Wilk’s lambda statistics) with the unstructured covariance matrix. Post hoc comparisons between pairwise time points were performed by contrast tests. All the assays were performed at a significance level of *α* = 0.05. In order to take into account the problem of multiple comparisons, along with original *p* values, the false discovery rate (FDR), adjusted *p* values were also calculated and reported in the text, where appropriate. Statistical analyses were conducted using SAS software, version 9.2 (SAS Institute, Cary, NC, USA).

## Results

### Course of the study

All the individuals completed the supplementation trial. There was also 100 % responsiveness to the questionnaires introduced at each time point of blood collection, and all the subjects reported to take Se yeast tablets regularly (1 tablet/day) for 6 weeks. Nobody declared smoking tobacco and taking Se containing supplements during the study. Dietary questionnaires concerned frequency of consumption of food products which contain the highest Se content in the Polish diet (according to our previous study [26]). As shown in supplementary figures (Figure S2), the overall consumption frequency of eggs, fish, and nuts was similar during 6-week period of supplementation as compared to the washout. Additionally, we collected data on factors (consumption of drugs, hormones, dietary supplements etc.) that could potentially affect results of the study (Table S2). Subjective side effects observed during supplementation, as reported by two female participants included short nausea directly after taking the supplement (one female) and vaginal thrush (the other female).

### Sex and genotype differences at baseline

Significant differences between males and females at baseline were observed for BMI (24.9 vs. 22.8, respectively, *p* < 0.0001) and GPx1 activity (16.1 vs. 18.2 U/g Hb, *p* = 0.01). Significant positive correlations with age were found in females for BMI (*ρ* = 0.34, *p* = 0.01), plasma Se or Sepp1 concentrations (*ρ* = 0.51, *p* = 0.0001; *ρ* = 0.39, *p* = 0.02, respectively), and TBARS (*ρ* = 0.43, *p* = 0.002). In males, positive significant correlations with age were observed only for TBARS (*ρ* = 0.54, *p* = 0.0002).

Numbers of male and female subjects within each genotype subgroup were similar (Table 2). There were no significant differences at baseline in terms of age, BMI, and Se status between the subjects with different *GPX1* genotype. *SEPP1* polymorphism was shown to significantly affect the baseline plasma Se concentration (in a linear manner), whereas no baseline differences were observed in terms of age, BMI, and plasma Sepp1 concentration.

### Se supplementation effects

Table 3 presents median values and a range of markers for selenium status (plasma concentrations of Se and Sepp1), oxidative stress (the activities of GPx1, GPx3, SOD1 and Cp, TAC, TBARS, and ROS generation), and DNA damage (at the level of DNA strand breaks and DNA oxidation) measured at baseline, during the supplementation trial and after 4 weeks of the washout period. Both plasma Se and Sepp1 median concentrations were significantly increased after 2 weeks of supplementation (98.00 µg/L vs. 62.65 µg/L and 7.36 vs. 3.86 ng/mL, respectively; *p* < 0.0001 and *p* < 0.0001, respectively) and after 6 weeks of supplementation (93.84 µg/L and 6.14 ng/mL, respectively; *p* < 0.0001 and *p* < 0.0001 vs. baseline, respectively). After 4 weeks of washout, Se started to decrease (74.61 µg/L, *p* < 0.0001 vs. 6 weeks); however, it was still significantly higher as compared to the baseline (*p* < 0.0001), whereas Sepp1 was not statistically different as compared to the median value observed after 6 weeks of supplementation (6.00 ng/mL, *p* = 0.270). GPx1 and GPx3 activities were significantly higher as compared to the baseline after 6 weeks of supplementation (24.43 vs. 17.10 U/gHb and 0.20 vs. 0.18 U/mL, respectively; *p* < 0.0001 and *p* < 0.0001, respectively), and both started to decrease slightly after 4 weeks of the washout period, though statistically significant difference as compared to the sixth week’s value was observed only for GPx3 (0.19 U/mL, *p* < 0.0001). Total plasma antioxidant capacity as well as lipid peroxidation was significantly increased already after 2 weeks of supplementation (*p* = 0.02 and *p* = 0.0002 vs. baseline, respectively) and remained significantly higher as compared to the baseline after 6 weeks of supplementation (*p* < 0.0001 and *p* < 0.0001, respectively) and after 4 weeks of the washout period (*p* < 0.0001 and *p* < 0.0001 vs. baseline, respectively). Plasma Cp activity was significantly increased after 6 weeks (*p* = 0.003 vs. baseline) and remained significantly higher as compared to the baseline after 4 weeks of washout (*p* = 0.01). SOD1 activity remained unaffected during the supplementation trial. The ability of whole-blood granulocytes to generate ROS upon PMA stimulation, measured only after 2 weeks of supplementation, was significantly decreased as compared to the baseline (*p* < 0.0001). A statistically significant increase in DNA damage, assessed at three time points, as compared to the baseline, was observed after 4 weeks of the washout period, both at the level of DNA strand breaks (*p* < 0.0001 vs. the baseline) and at the level of DNA oxidation (*p* < 0.0001 vs. baseline).

**Table 3:** Effect of Se supplementation on the studied biological parameters, including markers of selenium status, oxidative stress and DNA damage. Data presented regardless of genotype

Marker	Median and range, measured at four different time points				*p* value (MANCOVA)**
	Baseline	2 weeks	6 weeks	Washout	
Se (µg/L)	62.65^a^ (34.32–109.12)	**98.00** ^b^ (**56.49**–**183.86**)	**93.84** ^b^ (**54.92**–**148.76**)	**74.61** ^c^ (**49.27**–**111.58**)	*p* < **0.0001**
Sepp1 * (ng/mL)	3.86^a^ (0.86–36.03)	**7.36** ^b^ **(0.97**–**41.60)**	**6.14** ^c^ (**0.26**–**34.08**)	**6.00** ^c^ (**0.66**–**31.20**)	*p* < **0.0001**
GPx1 (U/gHb)	17.10^a^ (9.68–29.04)	16.24^a^ (8.93–28.17)	**24.43** ^b^ **(11.95**–**29.88)**	**23.76** ^b^ **(14.03**–**29.96)**	*p* < **0.0001**
GPx3 (U/mL)	0.18^a^ (0.12–0.25)	0.18^a^ (0.12–0.26)	**0.20** ^b^ **(0.14**–**0.28)**	**0.19** ^c^ (**0.12**–**0.26**)	*p* < **0.0001**
SOD1 (U/mg Hb)	6.20 (3.93–8.39)	6.12 (3.04–9.38)	6.02 (4.34*–*8.50)	6.00 (3.93–8.75)	*p* = 0.21
Cp (g/L)	0.46^a^ (0.24–0.91)	**0.49** ^b^ **(0.30**–**0.87)**	**0.48** ^b^ **(0.34**–**0.79)**	**0.49** ^b^ **(0.34**–**0.78)**	*p* = **0.01**
TAC * (mmol/L)	0.85^a^ (0.03–5.08)	**1.18** ^b^ **(0.10**–**4.99)**	**1.36** ^c^ **(0.05**–**5.25)**	**2.02** ^d^ **(0.24**–**5.95)**	*p* < **0.0001**
TBARS * (mmol/mL)	1.75^a^ (1.00–4.08)	**1.98** ^b^ **(1.02**–**4.24)**	**2.02** ^b^ (**1.02–4.52**)	**2.08** ^b^ (**1.01**–**3.29**)	*p* < **0.0001**
RFT (MFI index)	78.90^a^ (22.35 ± 134.12)	**67.66** ^b^ (**32.18** ± **113.57**)	na	na	*p* < **0.0001**
DNA strand breaks (% tail DNA)*	1.86^a^ (0.57–5.50)	na	1.91^a^ (0.63–3.90)	**2.10** ^b^ (**1.02**–**6.08**)	*p* < **0.0001**
DNA oxidation (% tail DNA)	2.58^a^ (0.00–5.74)	na	2.35^a,b^ (0.00–6.23)	**3.08** ^c^ (**0.00**–**6.49**)	*p* < **0.0001**

Statistically significant *p* values (last column) and values significantly different as compared to baseline are typed in bold. Values indexed with different upper case letters (a, b, c, d) are significantly different at *p* ≤ 0.05 (contrast test for different time points, adjusted for age, sex, BMI, and baseline plasma Se)

*na* parameter was not analyzed at this time point

* Skewed data; for these parameters, *p* values were calculated for log-transformed data

** Model adjusted for age, sex, BMI, and baseline plasma Se

Table 4 presents mean values of the normalized relative gene expression, analyzed at baseline, after 2 weeks of supplementation, after 6 weeks of supplementation, and after 4 weeks of the washout period. Five out of eight analyzed genes (*GPX1*, *GPX4*, *SEP15*, *SELS*, and *SELW*) were affected in a negative manner upon Se supplementation. Expression of mRNA for these genes was significantly decreased as compared to the baseline after 6 weeks of supplementation (*p* = 0.0002 for *GPX1*, *p* = 0.001 for *GPX4*, *p* < 0.0001 for *SEP15*, *p* < 0.001 for *SELS*, and *p* < 0.0001 for *SELW*) and remained significantly decreased as compared to the baseline after 4 weeks of the washout period (*p* < 0.0001 for *GPX1*, *p* = 0.0001 for *GPX4*, *p* < 0.0001 for *SEP15*, *p* < 0.0001 for *SELS*, and *p* < 0.001 for *SELW*). For two genes (*GPX4*, *SEP15*), a significant decrease as compared to the baseline was observed already after 2 weeks of supplementation (*p* = 0.006 and *p* = 0.02, respectively). *SEPP1* expression was the only case in which the expression was increased upon Se supplementation. However, the increase was not statistically significant as compared to the baseline, and it was significantly decreased after 4 weeks of the washout period (*p* = 0.001 and *p* = 0.0002 vs. 2 weeks and 6 weeks, respectively). Expression of the two genes, *TRXR1* and *SBP2*, remained unaffected.

**Table 4:** Selectively suppressive effect of selenium supplementation at the level of mRNA expression (relative expression, normalized to *GAPDH*). Data presented regardless of genotype

Gene	Mean and SD, measured at four different time points				*p* value, (MANCOVA)*
	Baseline	2 weeks	6 weeks	Washout	
*GPX1*	1.425 ± 0.212^a^	1.411 ± 0.187^a^	**1.355** ± **0.194** ^**b**^	**1.292** ± **0.208** ^**c**^	*p* < **0.0001**
*GPX4*	1.355 ± 0.217^a^	**1.294** ± **0.189** ^**b**^	**1.284** ± **0.193** ^**b**^	**1.251** ± **0.206** ^**c**^	*p* = **0.002**
*TRXR1*	1.742 ± 0.226	1.721 ± 0.197	1.748 ± 0.195	1.736 ± 0.208	*p* = 0.60
*SEPP1*	1.555 ± 0.292^a,c^	1.590 ± 0.316^a,b^	1.620 ± 0.284^a,b^	1.467 ± 0.318^a,c^	*p* = **0.001**
*SEP15*	1.625 ± 0.179^a^	**1.579** ± **0.181** ^**b**^	**1.526** ± **0.165** ^**c**^	**1.536** ± **0.223** ^**c**^	*p* < **0.001**
*SELS*	2.039 ± 0.274^a^	1.977 ± 0.245^a^	**1.916** ± **0.217** ^**b**^	**1.886** ± **0.309** ^**b**^	*p* < **0.0001**
*SELW*	1.220 ± 0.218^a^	1.183 ± 0.218^a,b^	**1.115** ± **0.213** ^**b**^	**1.125** ± **0.250** ^**b**^	*p* = **0.0003**
*SBP2*	1.470 ± 0.212	1.476 ± 0.183	1.472 ± 0.233	1.493 ± 0.210	*p* = 0.73

Statistically significant *p* values and mean values significantly different as compared to baseline expression are typed in bold. Values indexed with different upper case letters (a, b, c, d) are significantly different at *p* ≤ 0.05 (contrast test for different time points, adjusted for age, sex, BMI, and baseline plasma Se)

* Model adjusted for age, sex, BMI, and baseline plasma Se

### SNP alone effects and the interaction with Se supplementation

The effects of *GPX1* rs1050450 and *SEPP1* rs3877899 polymorphisms are presented in Table 5 and Table S3. The effects were analyzed regardless of time and with respect to time interaction to assess the possible modulatory SNP effects upon Se supplementation.

**Table 5:** Effects of *GPX1* rs1050450 and *SEPP1* rs3877899 polymorphisms on the markers of selenium status, oxidative stress and DNA damage, interaction with time

Parameter	*p* value for genotype effect (ANCOVA)* and for time interaction (MANCOVA)*		
	*GPX1* rs1050450	*SEPP1* rs3877899	*GPX1* rs1050450 × *SEPP1* rs3877899
Se (µg/L)	*p* = 0.94	*p* = 0.08	*p* = 0.36
Sepp1** (ng/mL)	*p* = 0.17	*p* = 0.13	*p* = 0.84
GPx1 (U/gHb)	*p* = **0.04** (Fig. 1a)	*p* = 0.33	*p* = 0.59
GPx3 (U/mL)	*p* = 0.47 Interaction with time: *p* = **0.05** (Fig. 2)	*p* = 0.33	*p* = 0.48
SOD1 (U/mg Hb)	*p* = 0.91^a^	*p* = 0.76 Interaction with time: *p* = **0.02** (Fig. 3 **)**	*p* = 0.64
Cp (g/L)	*p* = 0.20	*p* = 0.70	*p* = 0.33
TAC** (mmol/L)	*p* = 0.76	*p* = 0.37	*p* = 0.97
TBARS** (mmol/mL)	*p* = 0.09	*p* = 0.15	*p* = 0.65
RFT (MFI index)	*p* = 0.14	*p* = 0.95	*p* = 0.74
DNA strand breaks (% tail DNA)	*p* = 0.49 Interaction with time: *p* = **0.05** (Fig. 4)	*p* = 0.72	*p* = 0.47
DNA oxidation (% tail DNA)	*p* < **0.0001** (Fig. 5)	*p* = 0.62	*p* = 0.65

Statistically significant *p* values are typed in bold and supplied with graphical explanation on the indicated figure. For the interaction with time, only statistically significant *p* values are presented. Statistically nonsignificant effects are shown in the supplementary Figures S3a-q

* Models adjusted for age, sex, BMI, and baseline plasma Se

** Skewed data; for these parameters, *p* values were calculated for log-transformed data

#### Se status

Plasma Se and Sepp1 concentrations were not associated with *GPX1* rs1050450 (*p* = 0.94 and *p* = 0.17, respectively) or *SEPP1* rs3877899 (*p* = 0.08 and *p* = 0.13, respectively) nor with the combination of those two polymorphisms (*p* = 0.36, *p* = 0.84, respectively, Table 5). No modulatory effects were also observed for any of the two SNPs on the dynamic changes in Se status markers in response to Se supplementation (as assessed by the tests for interaction with time).

#### Oxidative stress and DNA damage

**Fig. 1:**
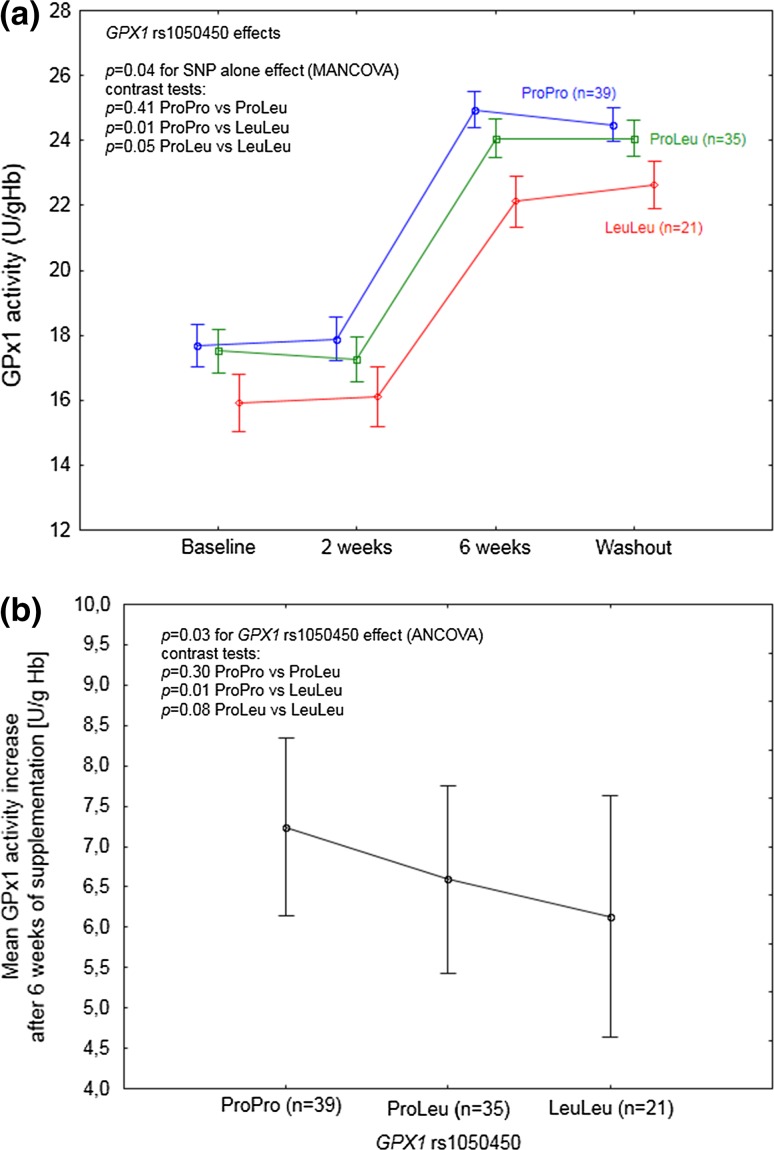
**a** *GPX1* rs1050450 effect on GPx1 activity in the individuals supplemented with selenium. A significant SNP effect was observed regardless of time. Data adjusted for age, sex, BMI, and baseline selenium; **b** mean GPx1 activity increase after 6 weeks of supplementation, with respect to *GPX1* genotype. Data adjusted for age, sex, BMI, and baseline GPx1 activity. *p* values for the ANCOVA/MANCOVA and contrast tests indicated in the figures

Statistically significant effects of *GPX1* rs1050450 on GPx1 activity (*p* = 0.04, Table 5) and DNA oxidation (*p* = 0.002; Table 5) were observed regardless of time (at each time point). According to the contrast tests, *GPX1* LeuLeu homozygotes were shown to differ significantly as compared to ProPro homozygotes and ProLeu heterozygotes, having the lowest GPx1 activity (Fig. 1a) and, at the same time, the highest level of DNA oxidation (Fig. 3). Additional analysis of mean GPx1 activity change after 6 weeks of Se supplementation with respect to *GPX1* rs1050450 genotype confirmed the significant impact of this SNP on enzyme activity response, showing that *GPX1* LeuLeu individuals were characterized by a significantly lower increase upon supplementation as compared to the individuals possessing ProPro genotype (*p* = 0.008; Fig. 1b).

**Fig. 2:**
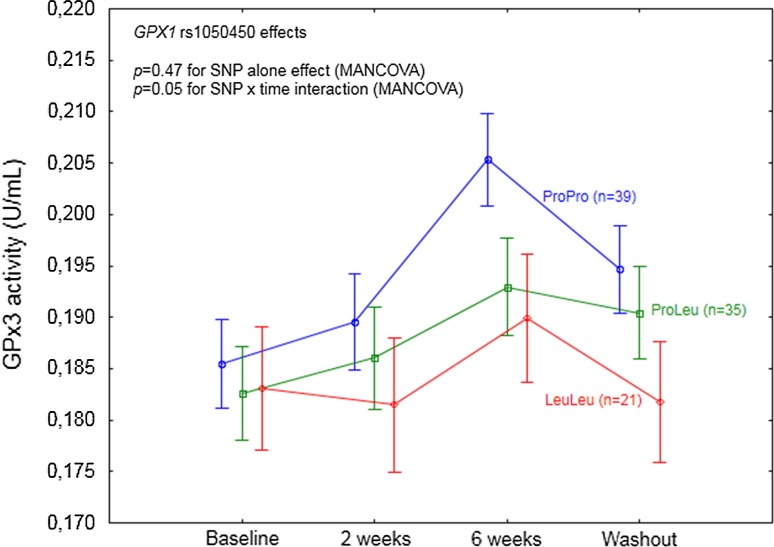
*GPX1* rs1050450 effect on GPx3 activity in the individuals supplemented with selenium. A significant SNP effect was observed in the interaction with time. Data adjusted for age, sex, BMI, and baseline selenium. *p* values for the MANCOVA indicated in the figure. According to the particular time points analysis, a significant difference in GPx3 activity as compared to baseline was shown after 6 weeks of supplementation and during the washout period for ProPro (*p* < 0.0001 and *p* = 0.002, respectively) and ProLeu (*p* = 0.001 and *p* = 0.02, respectively)

**Fig. 3:**
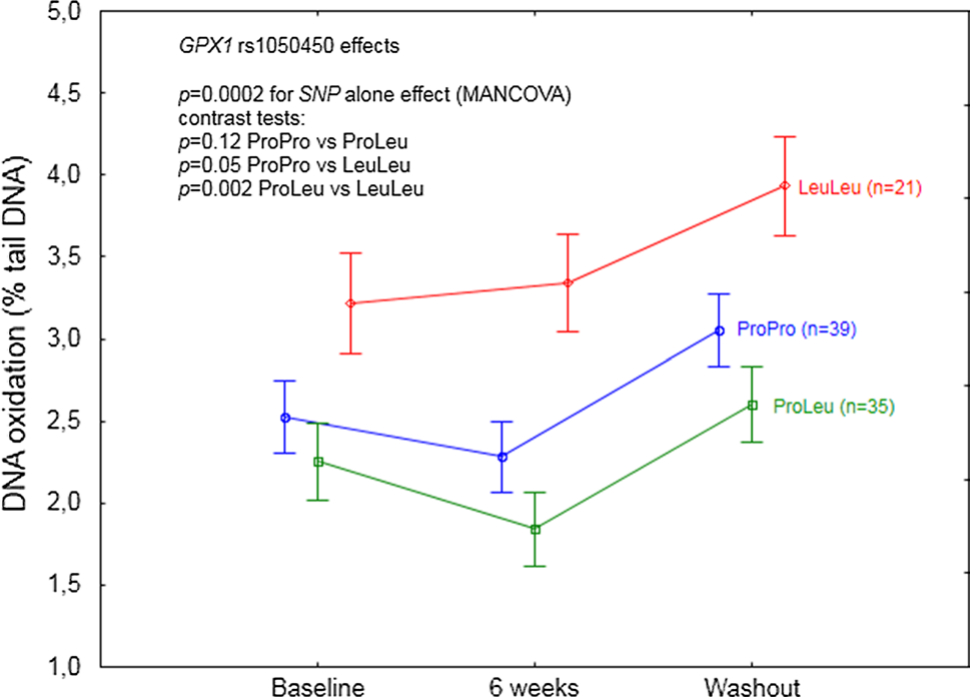
*GPX1* rs1050450 effect on DNA oxidation (expressed as % of DNA in comet tail) in the individuals supplemented with selenium. A significant SNP effect was observed regardless of time. Data adjusted for age, sex, BMI, and baseline selenium. *p* values for the MANCOVA and contrast test indicated in the figure

After inclusion of time effect (SNP and time interaction), *GPX1* rs1050450 was observed to modulate the response to Se supplementation at the level of GPx3 activity (*p* = 0.05; Table 5; Fig. 2). In the explorative analysis of particular time points, GPx3 response was shown to be affected by the genotype specifically after 6 weeks of supplementation and during washout, with significantly higher values as compared to the baseline observed for ProPro homozygotes (*p* < 0.0001 and *p* = 0.002 for 6 weeks and washout, respectively) and ProLeu heterozygotes *p* = 0.001 and *p* = 0.02 for 6 weeks and washout, respectively), whereas GPx3 activity in LeuLeu homozygotes was not affected (*p* = 0.21 and *p* = 0.66 for 6 weeks and washout, respectively).

**Fig. 4:**
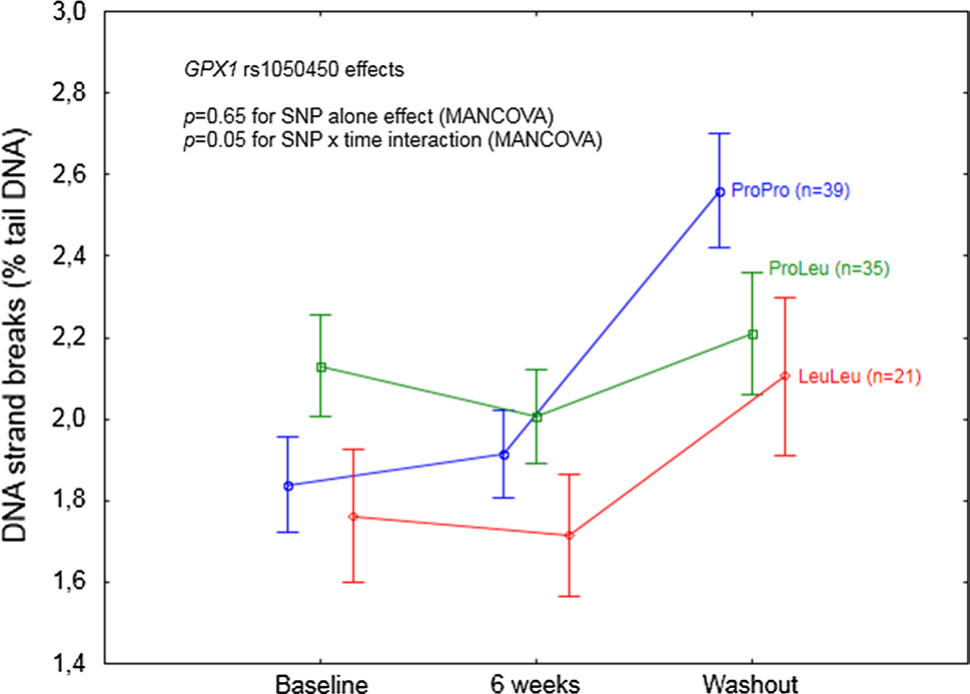
*GPX1* rs1050450 effect on DNA strand breaks (expressed as % of DNA in comet tail) in the individuals supplemeted with selenium. A significant SNP effect was observed in the interaction with time. Data adjusted for age, sex, BMI, and baseline selenium. *p* values for the MANCOVA indicated in the figure. According to the particular time point analysis, a significant difference in DNA strand breaks as compared to baseline was shown during washout for ProPro homozygotes, *p* < 0.0001

Statistically significant SNP and time interaction was shown for *GPX1* rs1050450 also at the level of DNA strand breaks (*p* = 0.05; Table 5; Fig. 4). According to the analysis of particular time points, a significant increase in DNA damage, as compared to the baseline, was shown at washout and only for ProPro homozygotes (*p* < 0.0001).

**Fig. 5:**
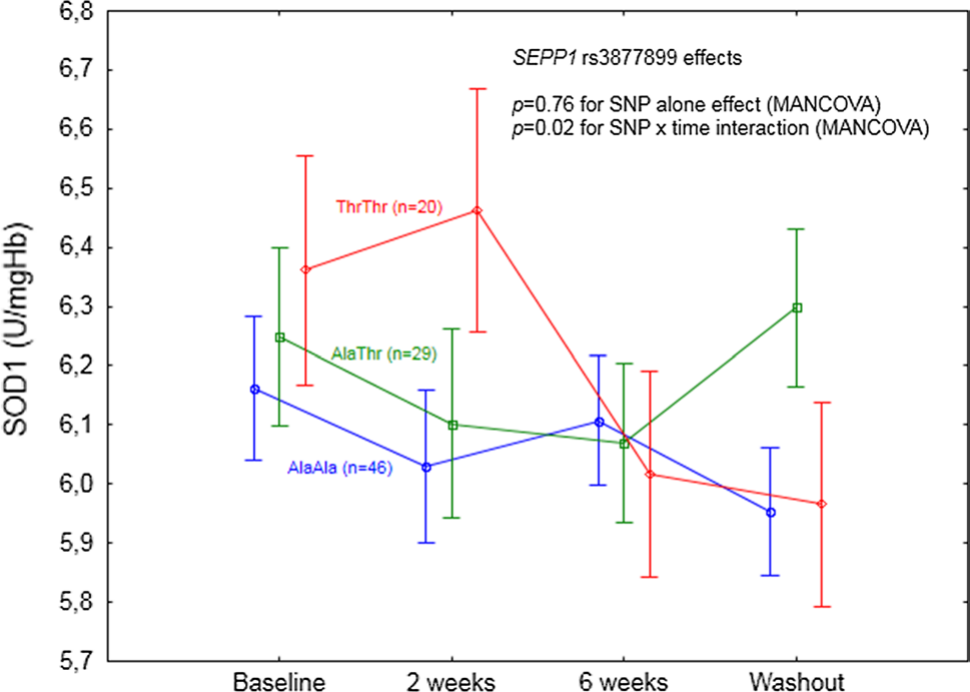
*SEPP1* rs3877899 effect on SOD1 activity in the individuals supplemented with selenium. A significant SNP effect was observed in the interaction with time. Data adjusted for age, sex, BMI, and baseline selenium. *p* values for the MANCOVA indicated in the figure. According to the particular time point analysis, a significant difference in GPx3 activity as compared to baseline was shown for AlaAla homozygotes during washout (*p* = 0.04)

Significant *SEPP1* rs3877899 effect was limited only to the response of SOD1 activity upon Se supplementation (*p* = 0.02 for SNP and time interaction, Table 5; Fig. 5). According to the analysis of particular time points, a significant SOD1 decrease, as compared to the baseline, was observed specifically for AlaAla homozygotes during washout (*p* = 0.04).

#### mRNA expression

*GPX1* rs1050450 and *SEPP1* rs3877899 SNPs were not associated with any changes in the expression of related (encoded) genes. *GPX1* rs1050450 was shown to modulate the expression of unrelated target, *SEP15*, upon Se supplementation, both alone (*p* = 0.03) and in combination with *SEPP1* rs3877899 (*p* = 0.04; Table S3).

### False discovery rate analysis

After FDR correction of *p* values, significant effects were maintained for all the analyzed data regardless of the genotype (Tables 3, 4), whereas most of the effects analyzed after genotype stratification were no more statistically significant (data not shown). The only genotype modulatory effect that remained statistically significant after FDR correction was the effect of *GPX1* rs1050450 polymorphism on DNA oxidation, regardless of time (FDR *p* values: 0.02).

## Discussion

### Final evidence that GPX1 Leu variant at rs1050450 is associated with a lower GPx1 response to Se

This supplementation trial, conducted among 95 non-smoking individuals, prospectively genotyped for two redox active selenoproteins, confirmed the previously observed effect of *GPX1* rs1050450 on GPx1 activity response to Se supplementation [24, 35]. Glutathione peroxidase is an important antioxidant enzyme, which is responsible for reducing hydrogen peroxide in the presence of reduced glutathione [36]. Although GPx1 is Se-dependent enzyme, its activity relies also on other different factors, including age, sex, smoking status, and health condition [37–39]. To exclude the modifying effect of some of these variables, the study included only the non-smoking individuals who reported themselves to be free from chronic diseases, whereas sex and age were included in the analysis as covariates. Functional effects linked to *GPX1* rs1050450 polymorphism, associated with proline (Pro)-to-leucine (Leu) substitution at codon 198 (or 200), were originally observed in MCF7 cells [35] and supported by some [27, 37, 40], though not all [41], human observational studies. Altogether, *GPX1* variant (Leu) allele has been suggested to cause lower responsiveness of GPx1 to Se. However, this hypothesis has not been entirely confirmed in human supplementation trials. Miller et al. [24] combined the results of two separate randomized, placebo-controlled double-blind trials (RCT), in which subjects (*n* = 255) with coronary artery disease were supplemented with 100 µg of Se (as SeMet) per day for 12 weeks. Average increase in GPx1 activity in the patients with at least one Leu allele was significantly lower as compared to ProPro homozygotes, but the difference between the genotypes was most evident in the subjects with the lowest baseline plasma Se concentration (83–99 µg/L). The second, smaller study was conducted among 37 obese women considered as Se-deficient (with the mean baseline plasma Se concentrations in different genotype groups ranging from 54 to 62 µg/L), who were supplemented with Brazil nuts for 8 weeks (providing 290 µg of Se/day) [25]. The authors have observed different magnitude of GPx1 activity increase depending on *GPX1* genotype; however, the difference was not statistically significant, possibly due to the low sample size (only five LeuLeu homozygotes). Our study, which was conducted among individuals with a relatively low baseline Se status (mean plasma Se 62.6 µg/L), provides final evidence, indicating that *GPX1* Leu allele at rs1050450 is associated with a lower GPx1 response to Se supplementation, specifically in the individuals with low Se dietary intake. This observation excludes the utility of GPx1 activity as a useful biomarker of Se status in supplementation trials, not only in the high Se status but also among the low Se status population, as previously suggested by us [27]. Furthermore, we also observed that *GPX1* rs1050450 polymorphism modulated the response of other peroxidase, GPx3, showing no enzyme responsiveness in the case of Leu homozygotes, after 6 weeks of supplementation. Because this SNP effect was not target related, the exact relationship remains unclear, though possibly resulting from strong redox-related interactions between both (erythrocyte and plasma) glutathione peroxidases.

### Unclear association between GPX1 polymorphism, Se supplementation, and DNA damage during washout

On the basis of their study, Miller et al. [24] have concluded that individuals possessing Leu allele(s) have a higher demand for Se compared to ProPro homozygotes, suggesting that they may especially benefit from Se supplementation in terms of a decreased risk of cancer or other diseases. This hypothesis, though being very promising in terms of personalized nutrition, has never been verified by human long-term supplementation trials, and furthermore, human gene association studies indicate for a rather unclear relationship between *GPX1* polymorphism and cancer (detailed review in [42]). Following the hypothesis considering antioxidant activity of selenoproteins as one of the anticancer mechanisms exerted by Se, it is surprising that only few authors have investigated the relationship between GPx1 activity and any disease risk in a prospective manner. To our knowledge, only two such studies have been conducted, indicating significant inverse correlations in breast cancer [43] or coronary artery disease [44]. Thus, it should be noted that even though the possible relationship between *GPX1* polymorphism and the risk of cancer may exist, there is no epidemiological evidence that GPx1 upregulation caused specifically by Se supplementation may decrease such a risk, and that such supplementation should be especially beneficial in *GPX1* LeuLeu individuals with a low Se status. Some insights into a possible beneficial effect of Se supplementation specifically in *GPX1* LeuLeu individuals may be obtained by the analysis of the relationship between *GPX1* rs1050450 polymorphism and cancer risk-related biomarkers, such as DNA damage, in the Se supplemented individuals. Such an analysis, so far, has been conducted only in two human supplementation trials. Caple et al. [22] analyzed the effect of *GPX1* polymorphism on DNA damage (as assessed by comet assay) in blood lymphocytes of 48 subjects receiving 100 µg of Se (in the form of antioxidant supplement containing also vitamins A, E, and C) for 6 weeks. No modifying effects of this SNP have been observed, either at the level of endogenous DNA damage, or at the level of peroxide-induced DNA damage (as expressed by percent of DNA in comet tail). In contrast, a significant effect of *GPX1* polymorphism at the level of DNA damage has been observed in Brazil obese women in the already mentioned study, indicating that after 8 weeks of Brazil nuts consumption, ProPro homozygotes (*n* = 18) had lower DNA damage (measured by comet length), as compared to the baseline. Surprisingly, LeuLeu homozygotes (*n* = 5) apparently did not benefit from the supplementation, having significantly higher DNA damage after 8 weeks as compared to the wild-type homozygotes [25]. In our study, we observed that Se supplementation had no effect on DNA damage (regardless of the baseline values, as assessed in the MANCOVA model) but, intriguingly, the levels of both DNA oxidation and DNA strand breaks were significantly increased as compared to baseline, after 4 weeks following discontinuation of supplementation. Notably, this specific time point was during (not after) washout, as both plasma Se and Sepp1 markers (being significantly increased by intervention) still remained high. LeuLeu homozygotes had a significantly higher level of DNA oxidation as compared to ProPro and ProLeu subjects, which could be linked to the lower GPx1 activity as compared to ProPro or ProLeu; however, these genotype differences in DNA oxidation were independent of time, clearly indicating that GPx1 increase caused by Se supplementation did not improve DNA repair specifically in LeuLeu homozygotes (nor did it in two other genotypes). Furthermore, considering DNA oxidation, the LeuLeu individuals seemed also to be the most negatively affected after supplementation discontinuation. In contrast, specifically ProPro homozygotes seemed to be the only genotype which was negatively affected by Se supplementation discontinuation at the level of DNA strand breaks, notwithstanding with the observation from the Brazil study (showing ProPro homozygotes to have decreased DNA damage after consumption of high Se content nuts) or with the observational study in New Zealanders in whom the lower DNA damage as a function of increasing serum Se level has been shown in ProPro homozygotes, but not in the ProLeu or LeuLeu subjects [23, 25]. Nevertheless, we failed to indicate that *GPX1* LeuLeu homozygotes may especially benefit from Se supplementation in terms of decreased DNA damage, which is consistent with the observed DNA damage effect in the LeuLeu subjects from the Brazil study [25]. Instead, we observed that *GPX1* polymorphism modulated a specific negative “withdrawal effect” linked to Se supplementation discontinuation.

The overall observation of increased DNA damage during washout (shown regardless genotype) was somewhat unexpected and points to unrecognized properties of Se that may be assigned to specific chemical forms of Se present in Se yeast. The composition of Se yeast largely depends on the supplier [45], and considering that different chemical forms of Se have different cytotoxic and biochemical effects [46], it may not be excluded that some forms of Se exert deleterious effects on genomic stability. Blessing et al. indicated for example that reducible Se compounds such as phenylseleninic acid, phenylselenyl chloride, selenocystine, ebselen, and 2-nitrophenylselenocyanate are able to affect zinc finger proteins involved in DNA repair [47].

### Se supplementation was associated with overlapped prooxidant and antioxidant effects, accompanied by selective suppression of selenoprotein mRNA expression

Considering daily dietary intake in Polish population (20–59 µg), the average daily intake of the element in this study during supplementation should have not exceeded the upper tolerable limits of 300 or 400 µg/day, set by the European Scientific Committee on Food or US Food and Nutrition Board [4, 48]; thus, no toxic effects were expected. Furthermore, post-supplementation plasma Se concentrations in the study individuals (55–149 µg/L) were close to or lower than those reported in plasma or serum in other short-term Se supplementation trials, such as SELGEN (109 µg/L, plasma), Danish study (126–154 µg/L, serum), New Zealand study (142.1 µg/L, plasma), or Brazil study (127–148 µg/L, plasma) [21, 24, 25, 49], and notably, it was even lower than presupplementation values observed in the long-term supplementation trials such as NPC (114 µg/L, plasma) or SELECT (135 µg/L, serum) [13, 50]. However, we observed that Se supplementation was associated with a significant increase in lipid peroxidation, which occurred already after 2 weeks of intervention and which did not diminish after 4 weeks of washout. This prooxidant effect was accompanied by the overall increased ability to challenge oxidative stress (as indicated by higher activities of GPx1, GPx3, Cp, and higher plasma antioxidant capacity), suggesting some overlapped antioxidant and prooxidant processes, possibly resulting from the overlapped activity of different Se compounds present in Se yeast [45]. At the same time, mRNA expression in white blood cells (WBC) for five selenoprotein-encoding genes (*GPX1*, *GPX4*, *SEP15*, *SELS*, and *SELW*) was significantly decreased, suggesting that such a negative transcriptional feedback could have been induced by excess of Se. In other words, additional intake of 200 µg of Se may be considered too high in the study group. Selenoprotein mRNA expression in WBC has been proposed as a potential marker of Se status [51, 52]. However, studies conducted in humans point to no correlation between selenoprotein gene expression in blood and plasma/serum Se in the subjects supposed to be already Se repleted or possessing relatively high baseline Se status [49, 52, 53]. In contrast, Pagmantidis et al. [54] have observed a subtle increase in mRNA expression for *SPS1*, *SEP15*, and *SELK* in the individuals with baseline plasma Se = 93.9 µg/L (*n* = 39,) who were supplemented with selenate for 6 weeks. The only study which is partially consistent with our results is a 12-week Se supplementation trial joined with trivalent influenza vaccination at week 10th. In the trial, 119 non-smoking men (age 50–64 years) with the mean plasma Se concentration of 95.5 µg/L were supplemented with Se in a form of Se yeast (50,100, or 200 µg/day) or Se-enriched onions (50 µg), and a significant decrease in mRNA expression has been observed after 10 weeks of supplementation (before vaccination) for *SELW* in the highest Se dose group (200 µg/day Se yeast) as compared to the placebo. For the two other analyzed selenoproteins (*SELS* and *SELR*), no changes in mRNA expression have been reported during Se supplementation (before vaccination) [55]. Notably, both *SELW* and *SELS* were decreased upon Se supplementation in our study. Altogether, the effect of Se intervention on mRNA selenoprotein expression in humans seems to be selective and possibly depends on the chemical form of Se (as supported by in vitro study [56]) as well as on the baseline Se status. Our study, which was conducted among the subjects with the lowest baseline Se status, as compared to other trials discussed indicated that a high dose intervention in the subjects with a relatively low dietary intake induced selective selenoprotein mRNA decrease. One cannot exclude that this negative transcriptional feedback resulted from a specific redox dysregulation linked to a too high difference in the redox state between presupplementation and (post)supplementation period, which consequently prevented the adaptation process to occur. This hypothesis seems in line with the new concept of reductive stress, which may be induced by upregulated GPx1 activity [42].

### Minor effect of SEPP1 rs3877899 polymorphism

The second SNP of interest in this study, *SEPP1* rs3877899, associated with the alanine (Ala) into threonine (Thr) change in the polypeptide chain at codon 234, was shown to affect expression of different Sepp1 isoforms, showing that variant (Thr) allele is associated with lower proportions of 60-kDa isoform, and that plasma Sepp1 concentration may depend on the interaction between this SNP and sex [21, 57]. In our study, we failed to find any effect of *SEPP1* polymorphism on Sepp1, either at the level of protein or mRNA. However, we observed that the SNP affected plasma Se concentration at baseline, with the lower levels in the subjects possessing Thr allele(s), and this effect was also observed during a supplementation trial at the level approaching statistical significance (*p* = 0.08). Meplan et al. have observed that *SEPP1* polymorphism modified the levels of plasma Se in a response to supplementation in BMI-dependent manner, suggesting that *SEPP1* may affect response to Se specifically in the individuals with higher BMI (higher than 30). In this study, as assessed by MANCOVA model, we failed to find such an interaction, possibly due to the small number of individuals with BMI higher than 30 (no BMI effects on plasma Se response were observed, either alone or in combination with *SEPP1* genotype, data not shown). Meplan et al. have observed several other, not target related, effects of *SEPP1* rs3877899 polymorphism, linked to the affected expression or activity of specific selenoproteins in blood compartments (TRxR1, GPx1, or GPx4). The authors have concluded that different expression of Sepp1 associated with *SEPP1* rs3877899 polymorphism may affect expression and activities of other selenoproteins by influencing Se supply to different tissues. In our study, such effects were minor and they concerned combined and time-dependent effect of *GPX1* and *SEPP1* polymorphisms on *SEP15* expression, which may reflect the complexity of interactions within human selenoproteome, pointing to its dependence on Se supply and Se-dependent redox regulation. The other *SEPP1* effect, observed in the interaction with time and linked to the activity of Se-independent antioxidant enzyme, superoxide dismutase 1, was rather not consistent and difficult to explain, though it may confirm some metabolic link between Sepp1 expression and redox pathway related to glutathione peroxidases as a product of SOD1 (hydrogen peroxide) is a substrate for GPx1 and GPx3.

## Study limitations

The major limitation of the study concerns multiple testing, which could have generated false associations. Indeed, after the false discovery rate analysis was conducted, most of the observed effects were no more statistically significant. However, the most significant effects (relationship between *GPX1* polymorphism and GPx1 activity as well as DNA oxidation) were in line with the previous hypotheses and observations. Other limitation of the study is associated with the fact that the study was not randomized as the subjects were selected according to the genotype, and the genotype distribution in the study group was not representative of the whole population. However, since we especially aimed at analyzing the effects of minor alleles, such selection seems to be justified. Finally, the reported results were not further confirmed by an independent replication analysis and need to be verified.

## Conclusions

Lack of beneficial effects of Se supplementation in the individuals with a relatively low Se status, as indicated in this study at the level of DNA damage, does not provide molecular basis for the hypothesis on anticancer activity of the element in the populations with its low dietary intake. Overall, our study confirmed the functional effect of *GPX1* rs1050450 polymorphism linked to the lower GPx1 activity response to Se supplementation; however, it did not support the hypothesis that individuals possessing *GPX1* variant allele(s) may especially benefit from the increased Se intake. Findings linked to the suppressed mRNA expression of selected selenoproteins and prooxidant effects observed during supplementation warrant further investigation, whereas observation of the increased DNA damage during washout may give further insights into the unclear relationship between DNA damage and Se [47, 58]. Intriguingly, the possible modifying effect of *GPX1* polymorphism on DNA damage during washout may shift the current interest of genetic profiling of selenoproteins from question “who may benefit from Se supplementation,” into “who may be less harmed by such a kind of intervention.”

## Electronic supplementary material

Below is the link to the electronic supplementary material.

Supplementary material 1 (TIFF 856 kb)

Supplementary material 2 (TIFF 62 kb)

Supplementary material 3 (TIFF 856 kb)

Supplementary material 4 (TIFF 856 kb)

Supplementary material 5 (TIFF 856 kb)

Supplementary material 6 (TIFF 856 kb)

Supplementary material 7 (TIFF 856 kb)

Supplementary material 8 (TIFF 856 kb)

Supplementary material 9 (TIFF 856 kb)

Supplementary material 10 (TIFF 856 kb)

Supplementary material 11 (TIFF 856 kb)

Supplementary material 12 (TIFF 856 kb)

Supplementary material 13 (TIFF 856 kb)

Supplementary material 14 (TIFF 856 kb)

Supplementary material 15 (TIFF 856 kb)

Supplementary material 16 (TIFF 856 kb)

Supplementary material 17 (TIFF 856 kb)

Supplementary material 18 (TIFF 856 kb)

Supplementary material 19 (TIFF 856 kb)

Supplementary material 20 (PDF 239 kb)
