# Supplementary figures and images for: DNA damage and oxidative stress response to selenium yeast in the non-smoking individuals: a short-term supplementation trial with respect to GPX1 and SEPP1 polymorphism
Source: Eur J Nutr. 2015 Dec 10;55(8):2469–84. doi: 10.1007/s00394-015-1118-4 (PMC5122617; doi:10.1007/s00394-015-1118-4)

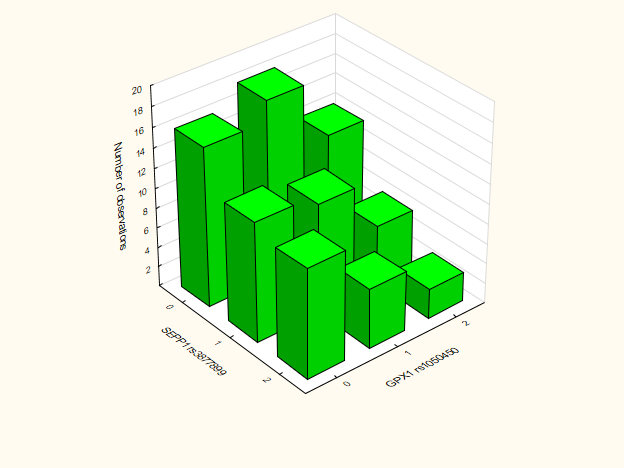

Supplement: Supplementary file 1 — Supplementary material 1 (TIFF 856 kb) [file 394_2015_1118_MOESM1_ESM.tif]

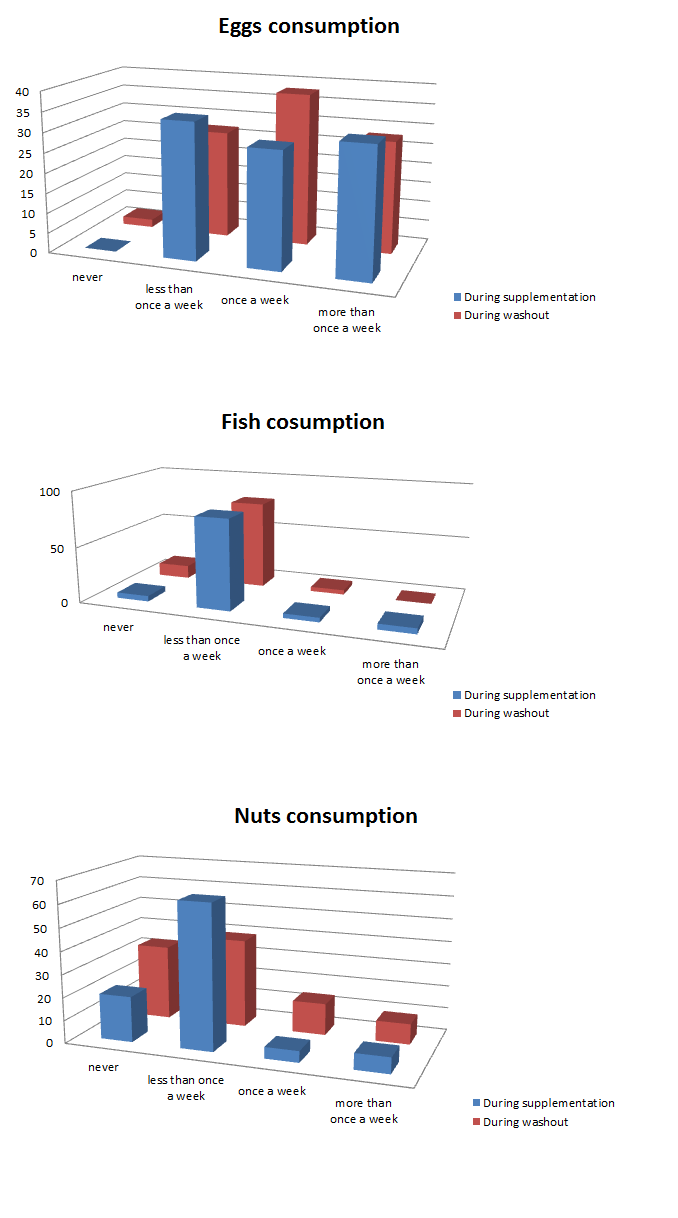

Supplement: Supplementary file 2 — Supplementary material 2 (TIFF 62 kb) [file 394_2015_1118_MOESM2_ESM.tif]

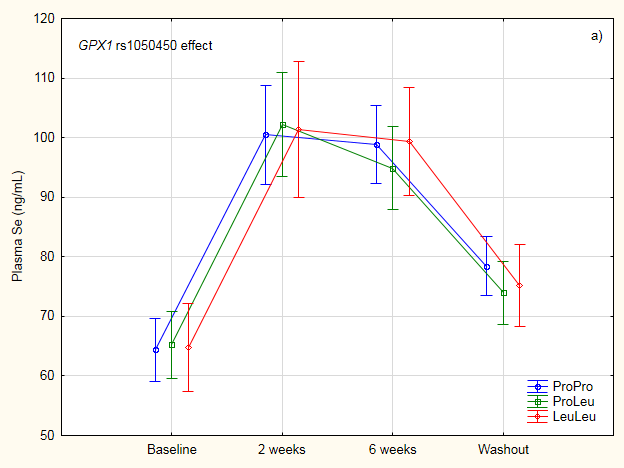

Supplement: Supplementary file 3 — Supplementary material 3 (TIFF 856 kb) [file 394_2015_1118_MOESM3_ESM.tif]

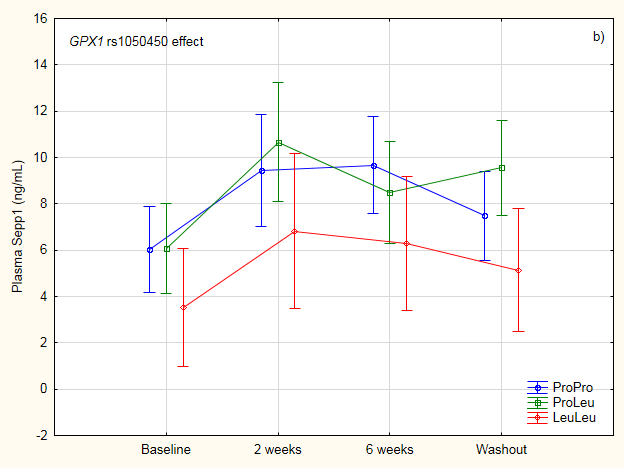

Supplement: Supplementary file 4 — Supplementary material 4 (TIFF 856 kb) [file 394_2015_1118_MOESM4_ESM.tif]

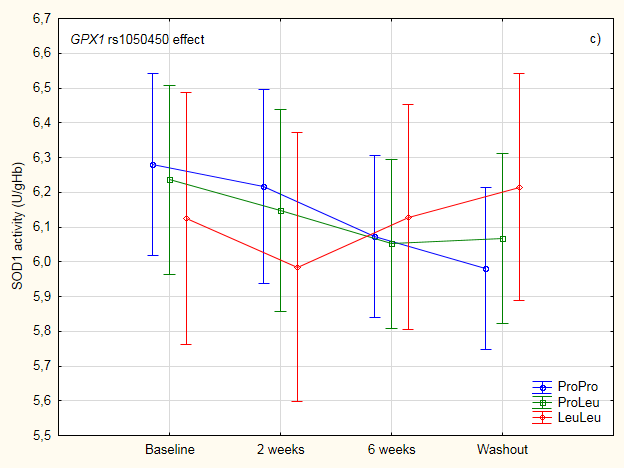

Supplement: Supplementary file 5 — Supplementary material 5 (TIFF 856 kb) [file 394_2015_1118_MOESM5_ESM.tif]

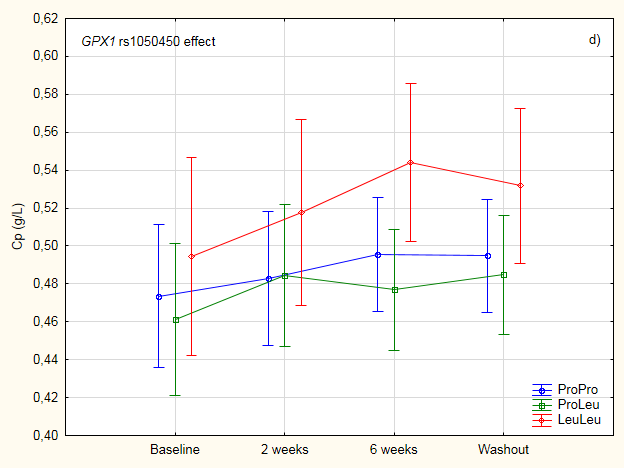

Supplement: Supplementary file 6 — Supplementary material 6 (TIFF 856 kb) [file 394_2015_1118_MOESM6_ESM.tif]

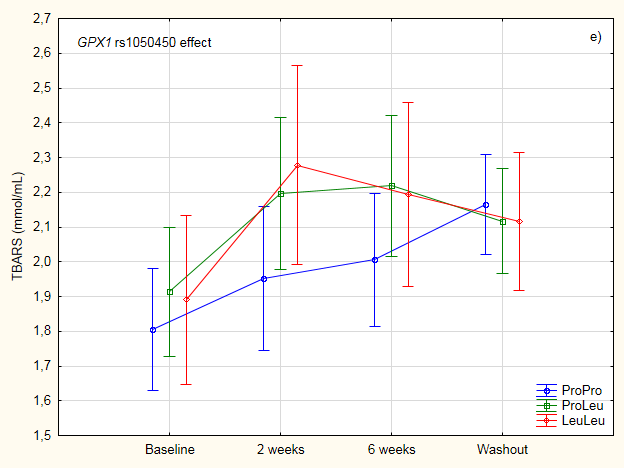

Supplement: Supplementary file 7 — Supplementary material 7 (TIFF 856 kb) [file 394_2015_1118_MOESM7_ESM.tif]

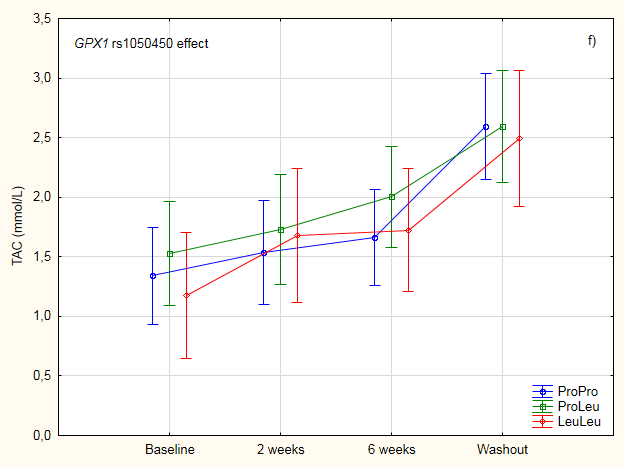

Supplement: Supplementary file 8 — Supplementary material 8 (TIFF 856 kb) [file 394_2015_1118_MOESM8_ESM.tif]

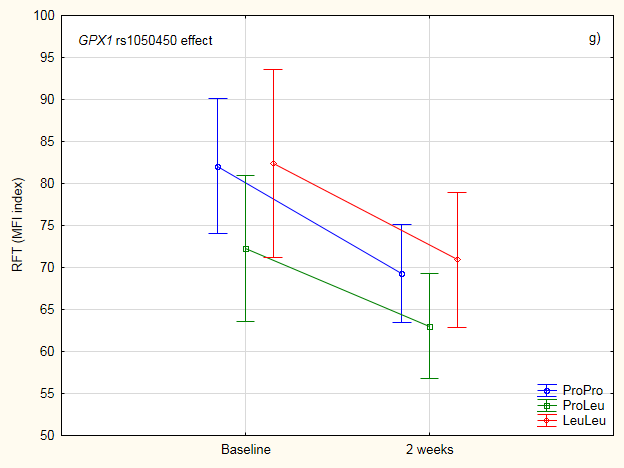

Supplement: Supplementary file 9 — Supplementary material 9 (TIFF 856 kb) [file 394_2015_1118_MOESM9_ESM.tif]

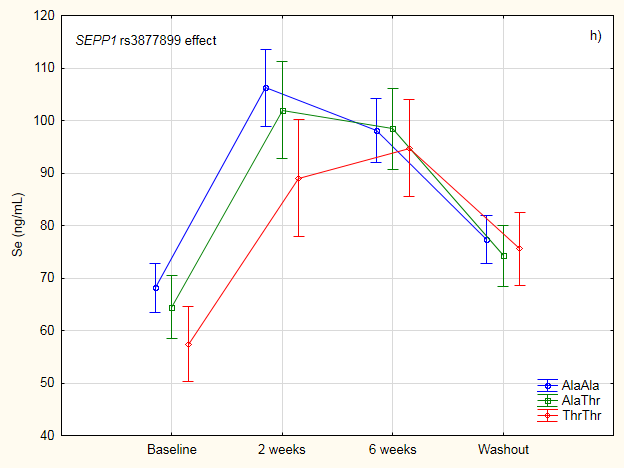

Supplement: Supplementary file 10 — Supplementary material 10 (TIFF 856 kb) [file 394_2015_1118_MOESM10_ESM.tif]

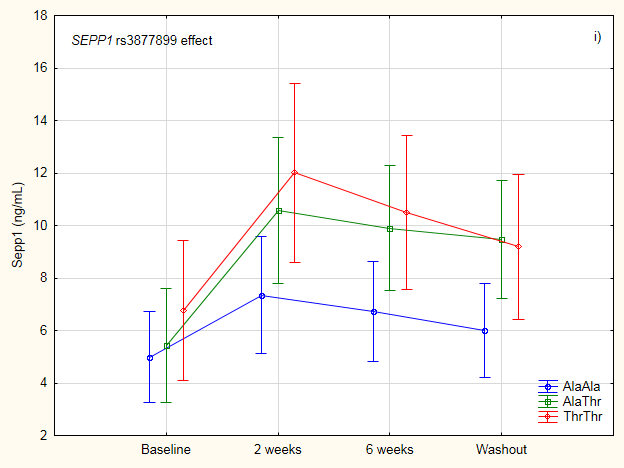

Supplement: Supplementary file 11 — Supplementary material 11 (TIFF 856 kb) [file 394_2015_1118_MOESM11_ESM.tif]

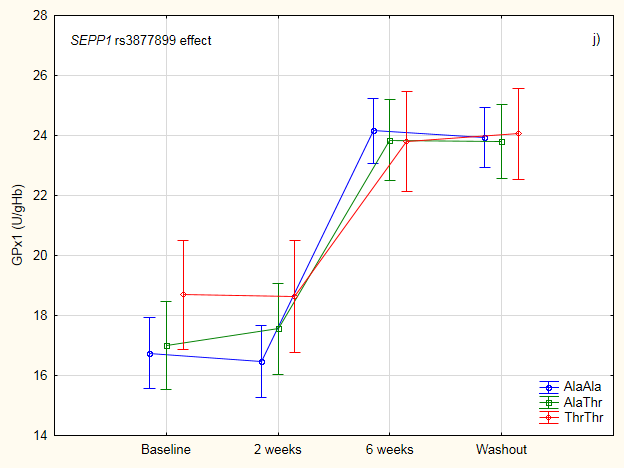

Supplement: Supplementary file 12 — Supplementary material 12 (TIFF 856 kb) [file 394_2015_1118_MOESM12_ESM.tif]

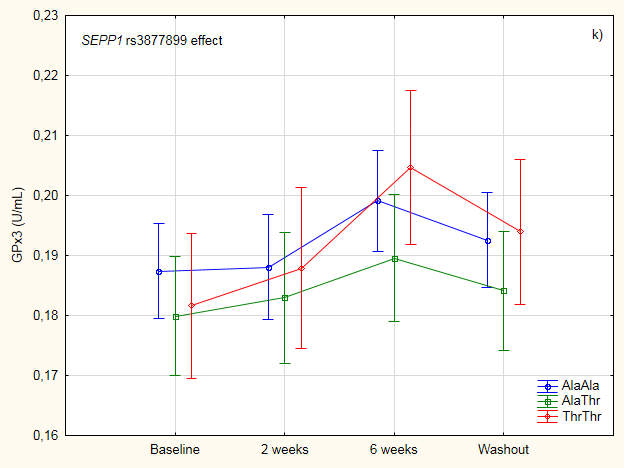

Supplement: Supplementary file 13 — Supplementary material 13 (TIFF 856 kb) [file 394_2015_1118_MOESM13_ESM.tif]

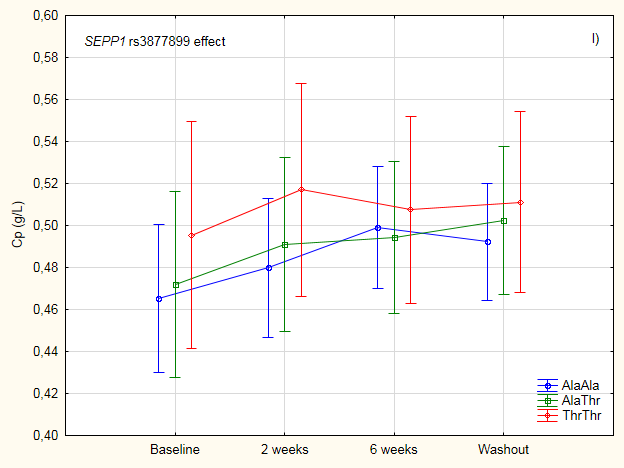

Supplement: Supplementary file 14 — Supplementary material 14 (TIFF 856 kb) [file 394_2015_1118_MOESM14_ESM.tif]

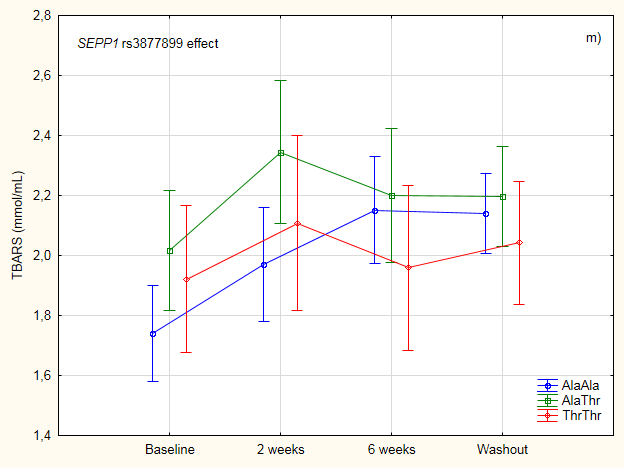

Supplement: Supplementary file 15 — Supplementary material 15 (TIFF 856 kb) [file 394_2015_1118_MOESM15_ESM.tif]

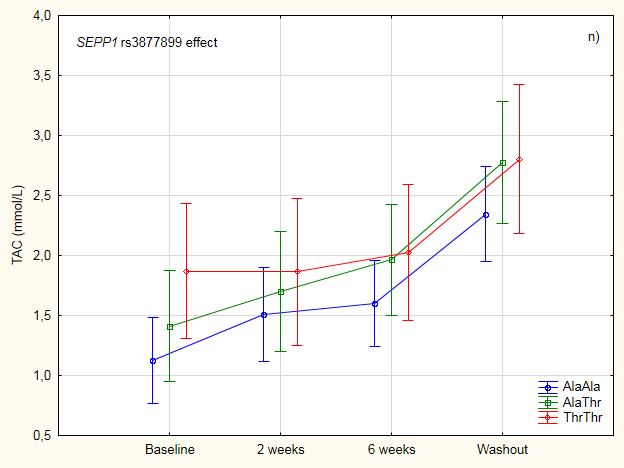

Supplement: Supplementary file 16 — Supplementary material 16 (TIFF 856 kb) [file 394_2015_1118_MOESM16_ESM.tif]

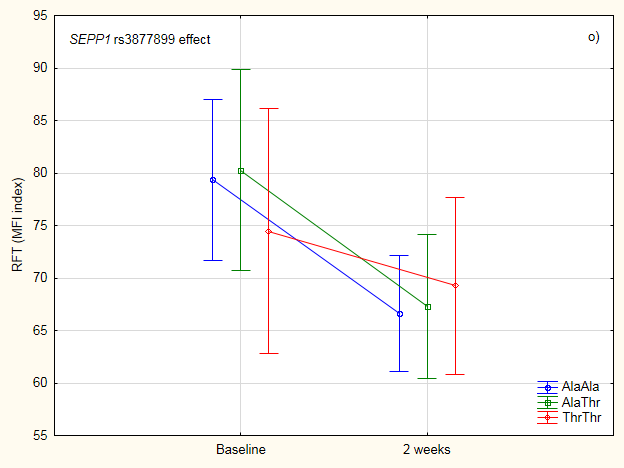

Supplement: Supplementary file 17 — Supplementary material 17 (TIFF 856 kb) [file 394_2015_1118_MOESM17_ESM.tif]

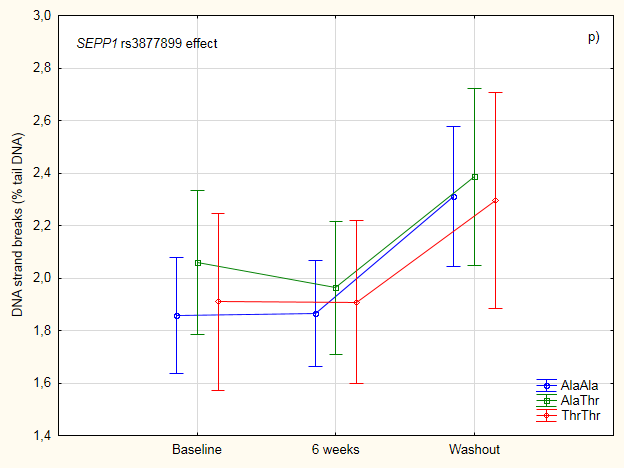

Supplement: Supplementary file 18 — Supplementary material 18 (TIFF 856 kb) [file 394_2015_1118_MOESM18_ESM.tif]

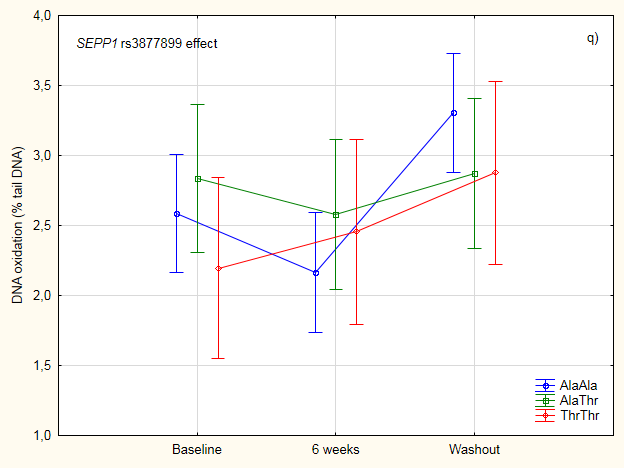

Supplement: Supplementary file 19 — Supplementary material 19 (TIFF 856 kb) [file 394_2015_1118_MOESM19_ESM.tif]
