# Supplementary material for: DNA damage and oxidative stress response to selenium yeast in the non-smoking individuals: a short-term supplementation trial with respect to GPX1 and SEPP1 polymorphism
Source: Eur J Nutr. 2015 Dec 10;55(8):2469–84. doi: 10.1007/s00394-015-1118-4 (PMC5122617; doi:10.1007/s00394-015-1118-4)
Supplement: Supplementary file 20 — Supplementary material 20 (PDF 239 kb) [file 394_2015_1118_MOESM20_ESM.pdf]

Supplementary materials (Table S1, Table S2, Table S3, Supplementary figure's legend)

Article title: DNA damage and oxidative stress response to selenium yeast in non-smoking individuals - short term supplementation trial with respect to *GPX1* and *SEPP1* polymorphism

Journal: European Journal of Nutrition

Authors: Jablonska E., Raimondi S., Gromadzinska J., Reszka E., Wieczorek E., Krol MB., Smok-Pieniazek A., Nocun M., Stepnik M., Socha K., Borawska MH., Wasowicz W.

Corresponding author:
Ewa Jablonska
Nofer Institute of Occupational Medicine, Department of Toxicology and Carcinogenesis, St. Teresy 8 Street, 91-348 Lodz, Poland
e-mail: ewa@imp.lodz.pl, Phone: +48 42 6314 612, Fax: +48 42 6356 83 31

Table S1. Nucleotide sequences of primers and products' length for Real Time PCR.

Gene	Gene product	NCBI accession numer	Starter F[1]	Starter R[1]	Amplicon size (nt)
GPX1	Glutathione peroxidase 1	NM_000581	caa cca gtt tgg gc tca g	gtt cac ctc gca ctt ctc g	121
GPX4	Glutathione peroxidase 4	NM_002085	ccc gat acg ctg agt gtg gtt tg	tta ctc cct ggc tcc tgc ttc c	76
SEPP1	Selenoprotein P	NM_005410	cag cct gga gca taa gag	cga gaa gag att cct tga tg	196
SEP15	15 kDa selenoprotein	NM_004261	ttg ggc tac ggt tgt tgt t	gca tgc ctc cga tga aaa	77
SBP2	SECIS binding protein	AF380995	atc tcc act cct tcg gtt g	gtc ggt aac aca agc atc c	180
SELS	Selenoprotein S	NM_203472	cgg atg agg cta aga atc ttg t	cag agc gct cct caa gag at	103
SEPW1	Selenoprotein W	NM_003009	ttg gct cag ggc taa tgc	ttc cta tca tga agc gtc tgc	82
TRXR1	Thioredoxin reductase 1	NM_003330	aca caa agc ttc agc atg tca	caa ttc cga gag cgt tcc	63
GAPDH	Glyceraldehyde 3-phosphate dehydrogenase	NM_002046	agc cac atc gct cag aca c	gcc caa tac gac caa atc c	66

[1] from 5' to 3'

Table S2. Questionnaire data collected from 95 selenium supplemented subjects during trial and during washout, concerning consumption of drugs, hormones, dietary supplements and herbs.

Type of consumption	Number of subjects (%)	
	During supplementation	During washout
Non-steroidal anti-inflammatory drugs	41 (43.2)	26 (27.4)
Antibiotics	11 (11.6)	4 (4.2)
Antihistamines	8 (8.4)	6 (6.3)
Statins	0 (0.0)	0 (0.0)
Hormones	8 (8.4)	6 (6.3)
Dietary supplements*	10 (10.5)	10 (10.5)
Sporadic use	8 (8.4)	8 (8.4)
Regular use	2 (2.1)	2 (2.1)
Selenium supplements	0 (0.0)	0 (0.0)
Herbs	14 (14.7)	9 (9.5)

* - multivitamins and/or minerals (excluding selenium)

Table S3. The effects of *GPX1* rs1050450 and *SEPP1* rs3877899 on mRNA expression and the interaction with time.

**Gene**	***p*-value for genotype effect (regardless of time, F test)* and for time interaction (MANCOVA)***		
	***GPX1* rs1050450**	***SEPP1* rs3877899**	***GPX1* rs1050450 x *SEPP1* rs3877899**
GPX1	*p*=0.37	*p*=0.96	*p*=0.95
GPX4	*p*=0.64	*p*=0.79	*p*=0.98
TRXR1	*p*=0.95	*p*=0.72	*p*=0.62
SEPP1	*p*=0.76	*p*=0.92	*p*=0.41
SEP15	*p*=1.00 **Interaction with time *p*=0.03**	*p*=0.96	*p*=0.58
SELS	*p*=0.87	*p*=0.98	*p*=0.63
SELW	*p*=0.74	*p*=0.91	*p*=0.72
SBP2	*p*=1.00	*p*=1.00	*p*=0.46

* models adjusted for age, sex, BMI and baseline plasma Se

Statistically significant *p*-values are typed in bold and supplied with graphical explanation on the assigned figure. For the interaction with time, only statistically significant *p*-values are presented

Supplementray figure's legend

Figure S1. Distribution of combined *GPX1* and *SEPP1* genotypes within study group (95 subjects). 0 – wild type homozygote, 1 – heterozygote, 2 – recessive homozygote.

Figure S2. Data from dietary questionnaires collected from selenium supplemented subjects, concerning consumption of food products with high Se content (eggs, fish and nuts) – during supplementation and during washout.

Figure S3. Nonsignificant effect of *GPX1* rs1050450 polymorphism on plasma Se (a), plasma Sepp1 (b), SOD1 activity (c), Cp activity (d), plasma TBARS (e), plasma TAC (f), and RFT (g), and nonsignificant effect of *SEPP1* rs3877899 polymorphism on plasma Se (h), plasma Sepp1 (i), GPx1 activity (j), GPx3 activity (k), Cp activity (l), plasma TBARS (m), plasma TAC (n), RFT (o), DNA stand breaks (p) and DNA oxidation (q). Relevant p-values indicated in Table 5.
